# Supplementary material for: Micheliolide Enhances Radiosensitivities of p53-Deficient Non-Small-Cell Lung Cancer via Promoting HIF-1α Degradation
Source: Int J Mol Sci. 2020 May 11;21(9):3392. doi: 10.3390/ijms21093392 (PMC7247679; doi:10.3390/ijms21093392)
Supplement: Supplementary file 1 [file ijms-21-03392-s001.pdf]

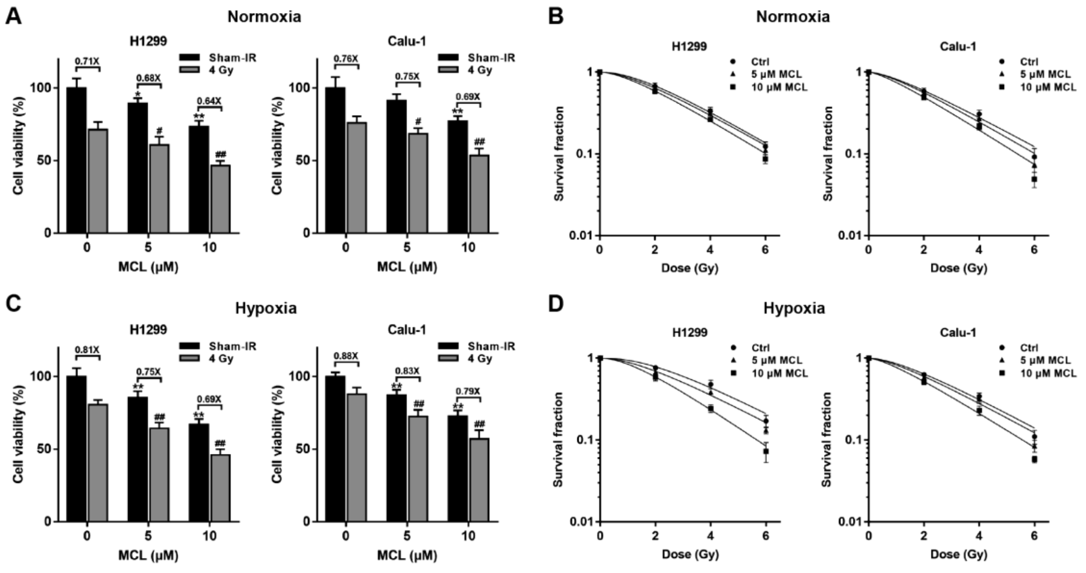

Figure S1. MCL sensitizes H1299 and Calu-1 cells to IR. (**A**) The relative cell viabilities of H1299 and Calu-1 cells were evaluated at 72 h after IR with or without MCL (5 or 10 μM) pretreatment under normoxia. *, p<0.05, **, p<0.01, compared with control (without radiation); $^{\#}$, p<0.05, $^{\#\#}$, p<0.01, compared with counterpart (without MCL treatment). (**B**) The survival curves of H1299 and Calu-1 cells after IR with or without MCL (5 or 10 μM) pretreatment under normoxia. (**C**) The relative cell viabilities of H1299 and Calu-1 cells were evaluated at 72 h after IR with or without MCL (5 or 10 μM) pretreatment under hypoxia. **, p<0.01, compared with control (without radiation); $^{\#\#}$, p<0.01, compared with counterpart (without MCL treatment). (**D**) The survival curves of H1299 and Calu-1 cells after IR with or without MCL (5 or 10 μM) pretreatment under hypoxia.

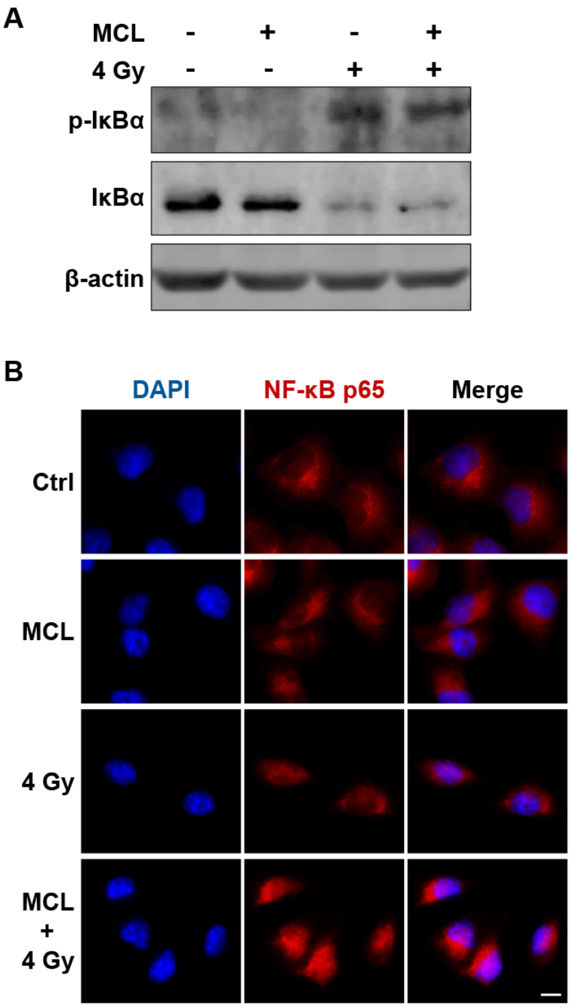

Figure S2. MCL treatment does not affect the IR-activated NF-κB signaling pathway in H1299 cells. (**A**) Western blot analysis of phosphorylation and degradation of IκBα at 2 h after IR in H1299 cells pretreated with or without MCL (20 μM). (**B**) Representative immunofluorescence images of intracellular localization of NF-κB p65 (red) in H1299 cells pretreated with or without MCL (20 μM) at 2 h after IR. Nucleus (blue) was stained with DAPI. Scale bar, 10 μm.

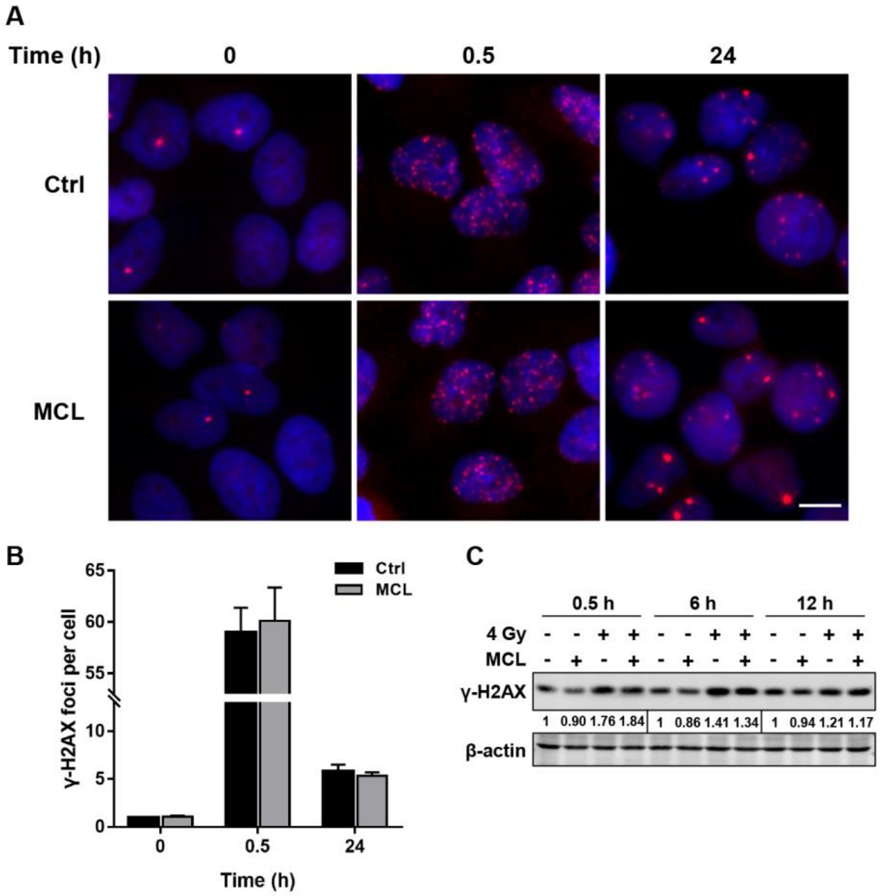

Figure S3. MCL does not affect the production and repair of DSBs after IR in H1299 cells. (**A**) Representative immunofluorescence images of γ-H2AX foci (red) in nucleus (blue) of H1299 cells at 0.5 and 24 h after IR (2 Gy) with or without MCL (20 μM) pretreatment. Scale bar, 10 μm. (**B**) Quantification of (**A**). (**C**) Expression of γ-H2AX protein at the indicated time points after IR (4 Gy) with or without MCL (20 μM) pretreatment in H1299 cells.

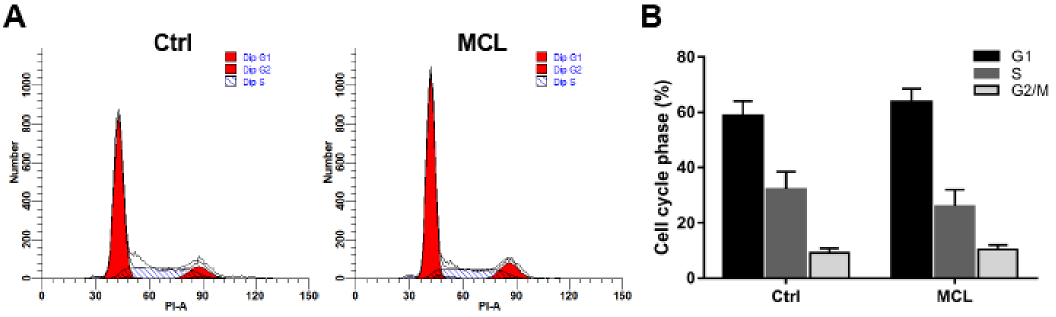

Figure S4. MCL does not affect the cell cycle redistribution of H1299 cells. (**A**) Cell cycle analysis of H1299 cells at 6 h following MCL (20 μM) treatment. (**B**) Quantification of (**A**).

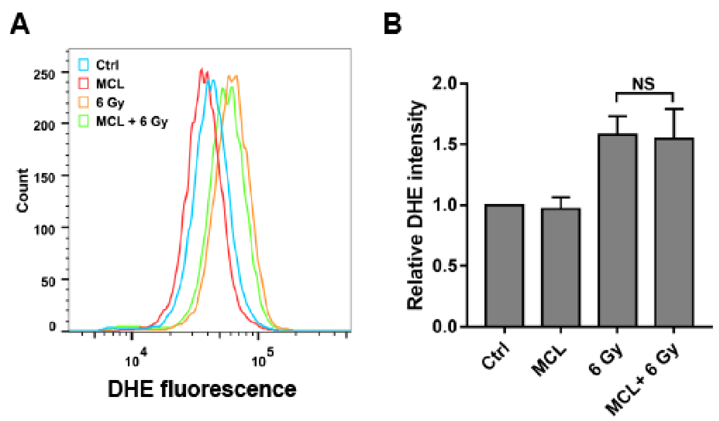

Figure S5. MCL does not affect the ROS production after IR in H1299 cells. (**A**) Determination of ROS with its specific fluorescent probe dihydroethidium (DHE) at 12 h after IR in H1299 cells pretreated with or without MCL (20 μM). (**B**) Quantification of (**A**).

Table S1. The survival curve parameters of H1299 and Calu-1 cells after IR with pretreatment of MCL (5 or 10 μM) under normoxia.

	H1299			Calu-1		
	**SF2**	**Dq**	**SERDq**	**SF2**	**Dq**	**SERDq**
Ctrl	0.67 ±0.06	1.75	-	0.58 ±0.05	1.13	-
5 μM MCL	0.63 ±0.07	1.47	1.18	0.55 ±0.04	1.03	1.10
10 μM MCL	0.58 ±0.04	1.26	1.39	0.49 ±0.02	0.76	1.48

[1] SF2, survival fraction at 2 Gy. [2] Dq, quasithreshould dose. [3] SERDq, sensitization enhancement ratio for Dq.

Table S2. The survival curve parameters of H1299 and Calu-1 cells after IR with pretreatment of MCL (5 or 10 μM) under hypoxia.

	H1299			Calu-1		
	**SF2**	**Dq**	**SERDq**	**SF2**	**Dq**	**SERDq**
Ctrl	0.76 ±0.03	2.37	-	0.63 ±0.03	1.42	-
5 μM MCL	0.68 ±0.04	1.75	1.36	0.59 ±0.05	1.17	1.21
10 μM MCL	0.59 ±0.06	1.39	1.71	0.51 ±0.04	0.90	1.59

[1] SF2, survival fraction at 2 Gy. [2] Dq, quasithreshould dose. [3] SERDq, sensitization enhancement ratio for Dq.

Materials and Methods

1. Immunofluorescence

Cells were fixed with 4% paraformaldehyde and permeabilised with TNBS solution (PBS supplemented with 0.5% Triton X-100 and 1% FBS) for 1 h. The cells were then incubated with specific primary antibodies for different proteins as follows: anti-γ-H2AX (phospho S139) antibody (1:200, Abcam) or anti-NF-κB p65 antibody (1:200, Cell Signaling Technology) at 37 °C for 2 h. After rinsing with TNBS, the cells were incubated with Goat Anti-Rabbit IgG H&L (TRITC) (1:1000, Abcam) at 37 °C for 1 h. DAPI (5 mg/mL; Sigma-Aldrich) was used to stain the nuclei. The cells were visualized with a fluorescence microscope (Leica DMI 4000B).

2. ROS measurement

ROS measurement was performed by flow cytometry in cells incubated with Dihydroethidium (DHE) (Molecular Probes) at a final concentration of 10 μM for 30 min at 37 °C.

3. Cell cycle analysis

Cells were harvested, fixed with 70% ethanol and stored at -20 °C for 24 h. Samples were then harvested by centrifugation at 1000 rpm for 5 min, washed twice with PBS and incubated with RNase A (0.25 mg/ml) and propidium iodide (20 μg/ml, BD Biosciences) for 30 min at 37 °C. Propidium iodide staining analysis was performed using a BD Accuri C6 analyzer (BD Biosciences). Cell cycle analysis was performed using Modfit LT 3.1 software.
